# Analysis of ambient temperature-responsive transcriptome in shoot apical meristem of heat-tolerant and heat-sensitive broccoli inbred lines during floral head formation

**DOI:** 10.1186/s12870-018-1613-x

**Published:** 2019-01-03

**Authors:** Chung-Wen Lin, Shih-Feng Fu, Yu-Ju Liu, Chi-Chien Chen, Ching-Han Chang, Yau-Wen Yang, Hao-Jen Huang

**Affiliations:** 1 Department of Life Sciences, National Cheng Kung University, No. 1, University Rd, Tainan City, 701 Taiwan; 2 Department of Biology, National Changhua University of Education, Changhua, 500 Taiwan; 3 Kale Biotech. Co, No.218, Fudong St., East Dist, Tainan City, 701 Taiwan; 4 Institute of Tropical Plant Sciences, National Cheng Kung University, No. 1, University Rd, Tainan City, 701 Taiwan

**Keywords:** Biomarker, Broccoli, Head formation, High temperatures, Genomic analysis

## Abstract

**Background:**

Head formation of broccoli (*Brassica oleracea var. italica*) is greatly reduced under high temperature (22 °C and 27 °C). Broccoli inbred lines that are capable of producing heads at high temperatures in summer are varieties that are unique to Taiwan. However, knowledge of the early-activated pathways of broccoli head formation under high temperature is limited.

**Results:**

We compared heat-tolerant (HT) and heat-sensitive (HS) transcriptome of broccoli under different temperatures. Weighted gene correlation network analysis (WGCNA) revealed that genes involved in calcium signaling pathways, mitogen-activated protein kinase (MAPK) cascades, leucine-rich repeat receptor-like kinases (LRR-RLKs), and genes coding for heat-shock proteins and reactive oxygen species homeostasis shared a similar expression pattern to *BoFLC1*, which was highly expressed at high temperature (27 °C). Of note, these genes were less expressed in HT than HS broccoli at 22 °C. Co-expression analysis identified a model for LRR-RLKs in survival-reproduction tradeoffs by modulating MAPK- versus phytohormones-signaling during head formation. The difference in head-forming ability in response to heat stress between HT and HS broccoli may result from their differential transcriptome profiles of *LRR-RLK* genes. High temperature induced JA- as well as suppressed auxin- and cytokinin-related pathways may facilitate a balancing act to ensure fitness at 27 °C. *BoFLC1* was less expressed in HT than HS at 22 °C, whereas other FLC homologues were not. Promoter analysis of *BoFLC1* showed fewer AT dinucleotide repeats in HT broccoli. These results provide insight into the early activation of stress- or development-related pathways during head formation in broccoli. The identification of the *BoFLC1* DNA biomarker may facilitate breeding of HT broccoli.

**Conclusions:**

In this study, HT and HS broccoli genotypes were used to determine the effect of temperature on head formation by transcriptome profiling. On the basis of the expression pattern of high temperature-associated signaling genes, the HS transcriptome may be involved in stress defense instead of transition to the reproductive phase in response to heat stress. Transcriptome profiling of HT and HS broccoli helps in understanding the molecular mechanisms underlying head-forming capacity and in promoting functional marker-assisted breeding.

**Electronic supplementary material:**

The online version of this article (10.1186/s12870-018-1613-x) contains supplementary material, which is available to authorized users.

## Background

Plants regularly encounter elevated temperature in their life cycle. Growth rates and developmental regulation greatly differ in response to temperature ranging from 12 to 27 °C [1]. Flowering time, which is heavily influenced by the environmental cues, is a key step in the life cycle of plants. The mechanisms underlying flowering-time control during vernalization are well studied [2, 3]. However, knowledge of how plants control flowering-time in response to high temperature remains elusive. Broccoli (*Brassica oleracea var. italica*) is a highly nutritious vegetable crop that contains high concentrations of vitamins, minerals, and anti-cancer substances (such as sulforaphane and glucosinolate) [4, 5]. The optimal temperature for broccoli growth is 18 °C or below [6]. These relatively low-temperature conditions are necessary to induce vernalization and allow for normal flower and head development [6]. Higher temperatures (30 °C) cause uneven-sized flower buds on broccoli inflorescences [7].

Flowering plants have evolved a complex network of regulatory mechanisms to ensure the proper timing of reproductive transition. Transcriptional regulation of gene expression in the vernalization pathway plays an important role in Brassicaceae [8, 9]. Induction of flowering occurs in response to several weeks of cold conditions but not after a few days of cold such as a temporary cold spell in autumn. The central flowering regulator FLOWERING LOCUS C (FLC) antagonistically regulates downstream flowering-related genes [10, 11]. As a MADS-box transcription factor (TF), FLC inhibits expression of the downstream components SUPPRESSOR OF OVEREXPRESSION OF CO 1 (SOC1) and FLOWERING LOCUS T (FT) [12, 13]. The repression of these genes delays the expression of the floral meristem identity genes APETALA1 (AP1) and LEAFY (LFY) to prolong vegetative stages [14, 15]. In addition, allelic variants of FLC are associated with variation of flowering time [15]. In the early developmental stages of *Arabidopsis*, *FLC* is highly expressed and its expression is modulated dynamically by various regulators, including vernalization, DNA methylation, and histone acetylation within the promoter-transcription start region [16, 17].

Basal thermotolerance in plants refers to the ability to tolerate elevated temperature, whereas adaptive capacity to survive under lethal high temperature after pre-exposure to sub-lethal temperature is defined as acquired thermotolerance [18, 19]. The early sensing of mild temperature upshift (22 °C to 30 °C) has been reported to occur at the plasma membrane of plant cells [20]. Higher temperature increases membrane fluidity and generates a significant heat shock response (HSR). Secondary messengers, such as calcium and hydrogen peroxide, have been reported to be involved in HSR [21, 22]. Heat shock proteins (HSPs) are important in thermotolerance reactions and act as molecular chaperones to prevent the denaturation or aggregation of proteins [23, 24]. Overexpression of the *Brassica campestris HSP70* gene in tobacco enhanced thermotolerance and increased superoxide dismutase (SOD) and peroxidase (POD) activities [25].

To cope with various stresses using limited resources, plants have evolved diverse mechanisms that enable the allocation of resources to address the most life-threatening stress. The trade-offs between responses to different stresses and growth regulation in plants are often regulated by crosstalk between signaling pathways [26, 27]. Signaling components such as phytohormones [28, 29], reactive oxygen species (ROS) [30], and Ca^2+^ [31] have been implicated in the crosstalks that mediate the trade-offs between plant growth and stress responses.

Glucosinolate produced by vegetables such as broccoli and cabbage provide anticarcinogenic and antioxidative activity [32, 33]. Glucosinolate is also an amino acid-derived compound responsible for defense against pathogens [34]. In *Arabidopsis halleri*, a negative correlation was found between total glucosinolate concentration and zinc hyperaccumulation in the leaves [35]. Thus, understanding the mechanisms that govern trade-offs between growth and response to stresses might provide important clues for plant breeders and researchers for producing crop plants.

Although the genetic regulation of flowering on *Arabidopsis* is well understood, less is known about the temperature regulation of broccoli floral head formation. The heat-tolerant (HT) broccoli inbred lines that can produce heads at high temperature in summer are unique to Taiwan [36]. HT broccoli genotypes were selected for their low vernalization requirement. In this study, we used a transcriptome-based analysis to explore expression differences of HT and HS broccoli genotypes. We examined the expression of genes responsible for flowering regulation, signaling pathways, heat shock regulation, ROS homeostasis, and the glucosinolate metabolic process of the two broccoli genotypes under high temperatures. The genes involved in regulatory networks of phytohormones may be associated with head formation in HT. Our results provide evidence that *BoFLC1* might be a useful molecular marker for plant breeding via marker-assisted selection.

## Results

### Whole genome co-expression analysis of HT and HS broccoli at different temperatures by weighted gene correlation network analysis (WGCNA)

**Fig. 1:**
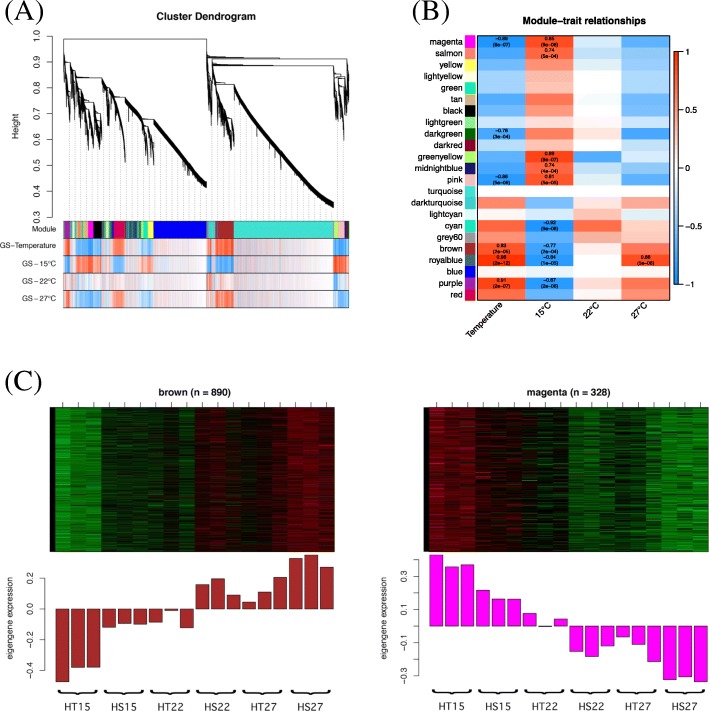
Weighted gene co-expression network analysis (WGCNA) of temperature-associated genes in the heat-tolerant and heat-sensitive broccoli genotypes. **a** Clustering dendrogram of genes showing module membership in colours. The y axis represents network distance as determined by 1 - topological overlap (TO), where values closer to 0 indicate greater similarity of probe expression profiles across samples (experimental treatments). Bottom: the first band shows module membership in colours. Additional bands indicate positive (red) and negative (blue) correlation to 15 °C, 22 °C, and 27 °C (see scale bar in B). **b** Colours to the left represent the 14 modules in the gene co-expression network. For each module, the heatmap shows module eigengen (ME) correlations to traits (4 groups of experimental treatment). Numbers in each cell report the correlation coefficients and Student asymptotic *P* value (parentheses) for significant ME-trait relationship. Scale bar, right, indicates the range of possible correlations from positive (red, 1) to negative (blue, − 1). **c** Relationship between the WGCNA modules (brown and magenta) and the three different temperatures (upper panel) and expression of the corresponding eigengene across the samples in the modules (lower panel). The heatmap (upper panel) and barplot of eigengene expression (lower panel) have the same samples (x axis). Rows of the heatmap correspond to genes, columns to samples; red in the color key denotes overexpression, green underexpression

To reveal the difference in the signaling pathway between the HT and HS genotypes under different temperatures (15 °C, 22 °C, and 27 °C), we performed microarray analysis of shoot meristems from the HT and HS genotypes by using the *Brassica napus* microarray chip (Agilent, Cat. No. G2519F-022520). Shoot meristems were collected at 50 days post-germination (DPG) before head formation. Both HT and HS genotypes exhibited head-forming capacity at 15 °C, but only the HT genotype showed head-forming capacity at 22 °C and 27 °C [37]. Thus, 22 °C and 27 °C were defined as high temperature treatments of the broccoli plants. After data were normalized by using GeneSpring v12 (Agilent, USA) and filtered by fold change and false discovery rate (FDR)-adjusted *P* value, 13,830 probes were included. Gene expression profiles of the microarray data were analyzed by using WGCNA [38] to identify gene co-expression patterns that might play roles in response to different temperature regimes. A total of 23 modules were found after setting a minimum module size of 2 (Fig. 1a and b). To determine whether any of the 23 modules were associated with the observed floral development profiles at different temperatures, we tested the correlations of the module eigengenes (MEs) with the differences between the temperature treatments (i.e., temperature trait). Six modules were found significant at the defined cut-offs (Bonferroni correction, significance threshold = 0.05). Brown, royal-blue, and purple modules were positively correlated with the temperature trait, whereas magenta, dark-green, and pink modules were negatively correlated (Fig. 1b). To examine the transcript response related to the temperature treatment, we depicted the expression of genes across all samples for these modules (Fig. 1c and Additional file 1: Figure S1). The eigengenes of the brown module showed higher expression at 22 °C and 27 °C in the HS (mean = 0.15 and 0.32) than HT genotype (mean = − 0.07 and 0.12) (*P* = 0.008 and 0.03, t-test), whereas the eigengenes of the magenta module showed higher expression at 15 °C in HT (mean = 0.39) than HS (mean = 0.18) (*P* = 0.002, t-test).

**Fig. 2:**
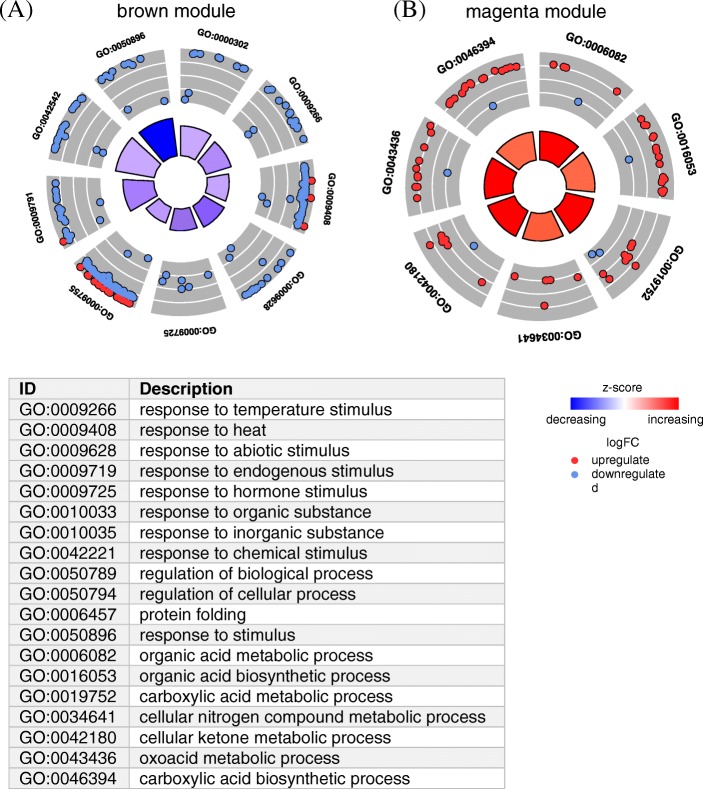
Gene Ontology (GO) enrichment analysis of WGCNA gene modules. GO circle plot displaying gene-annotation enrichment analysis. **a** The brown module. **b** The magenta module. Radar chart shows the distribution of individual terms in the annotation categories. The expression profiles were normalized with the RMA algorithm based on median baseline. The fold changes (FC) of gene expression values (log_2_ FC) were derived from 3 biological replications corresponding to each sample. Within each selected GO term, blue dot shows a gene downregulated at 22 °C (HT/HS) and red dot indicates a gene upregulated at 22 °C (HT/HS). The outer to inner layers of gray circles indicate the relative fold-change of gene expression (from higher to lower). The height of the inner rectangle represents the *P* value of the GO term. The rectangle is coloured with the blue-red gradient according to the z score. (*P* < 0.05, FDR adjusted *P* < 0.05) Z-score = (upregulated – downregulated) /$\sqrt{\mathrm{upregulated}+\mathrm{downregulated}}$

To reveal the biological processes (BP) underlying the transcriptome, we performed a GO enrichment analysis for detecting significantly overrepresented GO categories in each WGCNA module, by using the agriGO database [39]. These plots show a circular representation of the relative fold changes of gene abundances in HT compared to the genes in HS at 22 °C. The enriched BP GO terms (*P* < 0.05, FDR adjusted *P* < 0.05) in the brown module include “response to temperature stimulus”, “response to heat”, “response to abiotic stimulus”, and “response to hormone stimulus” (Fig. 2 and Additional file 2: Table S1). The enriched BP categories in the magenta module include “organic acid metabolic process”, “organic acid biosynthetic process”, “cellular nitrogen compound metabolic process”, and “carboxylic acid biosynthetic process”.

**Fig. 3:**
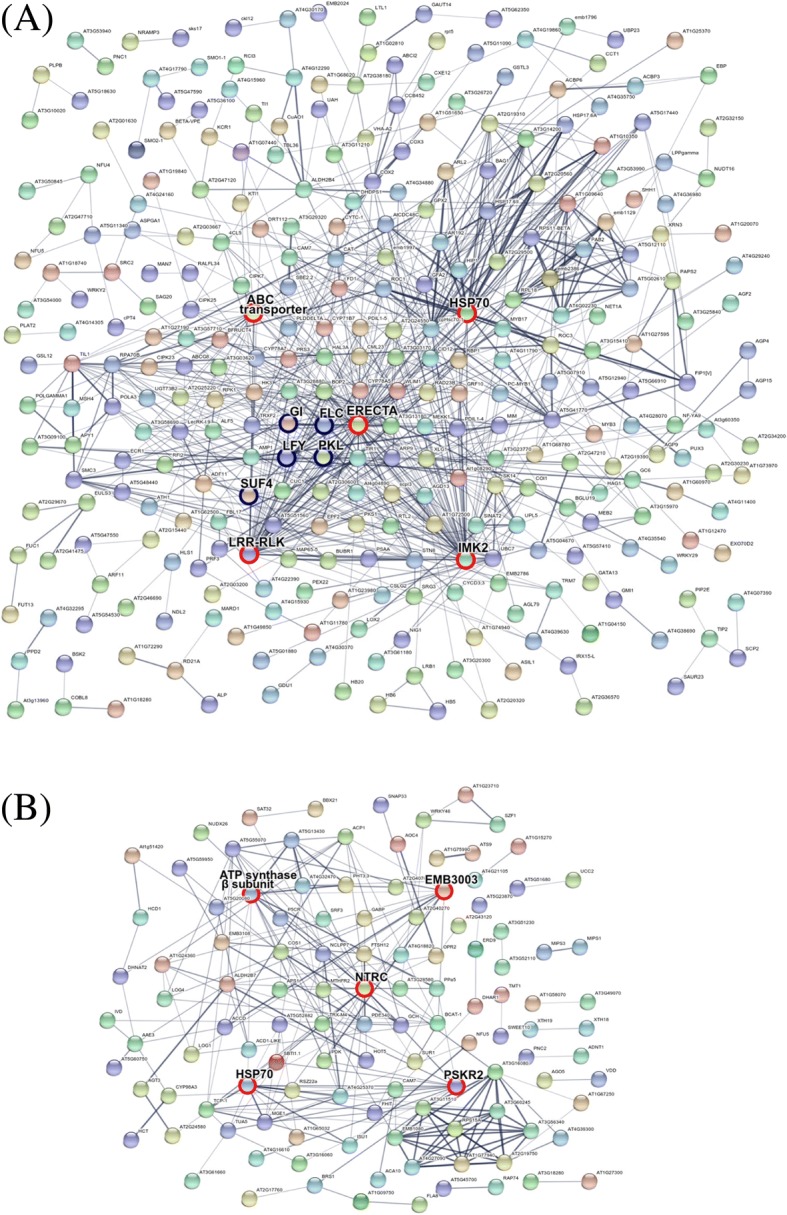
Network component analysis for the proteins within the WGCNA modules. Genes belonging to the brown (**a**) or magenta (**b**) modules were used as a query in the STRING database (http://string-db.org). After excluding proteins without interaction, the visualization network contained 114 nodes (genes) and 188 edges (connections). The hub proteins are marked by circles lines. Red indicates the top 5 connection proteins; Blue indicates the proteins involved in floral development

To gain insight into the functional interaction network, we used the STRING system (http://string-db.org), which functionally links proteins based on predictions, neighborhood analysis, experimental results, and literature mining. Probe lists of the brown and magenta modules were further filtered such that only probes with associated annotation were included for network analysis. The protein-protein interactions among the brown and magenta modules were detected on the basis of the minimum required interaction score set as 0.7 (high confidence) to remove proteins without connections (Fig. 3 and Additional file 3: Table S2). The hub genes within the brown module are ERECTA (67 interactions), IMK2 (55 interactions), LRR-RLK (leucine-rich repeat receptor-like kinase) (54 interactions), HSP70 (34 interactions), and ABC transporter (31 interactions) (Fig. 3a). ERECTA, IMK2, and LRR-RLK belong to the LRR-RLK family [40, 41]. The highly connected genes within the magenta module are NTRC (34 interactions), HSP70 (18 interactions), ATP synthase β-subunit (15 interactions), PSKR2 (14 interactions), and EMB3003 (12 interactions) (Fig. 3b). NTRC is a NADPH-dependent thioredoxin reductase involved in plant protection against oxidative damage [42]. PSKR2 encodes phytosulfokine receptor 2 [43], and EMB3003 is embryo defective 3003 [44]. Floral development-related genes in the brown module included FLC, LFY, GI and SUF4 (Fig. 3a). Of note, ERECTA, FLC, LFY, GI and SUF4 were highly connected. Furthermore, in these significant modules associated with temperature, we only identified the *FLC*-associated genes presented in the brown module. Hence, genes involved in the brown module might be associated with floral development of broccoli in response to high temperature.

### Expression of BoFLCs in HT and HS broccoli

**Fig. 4:**
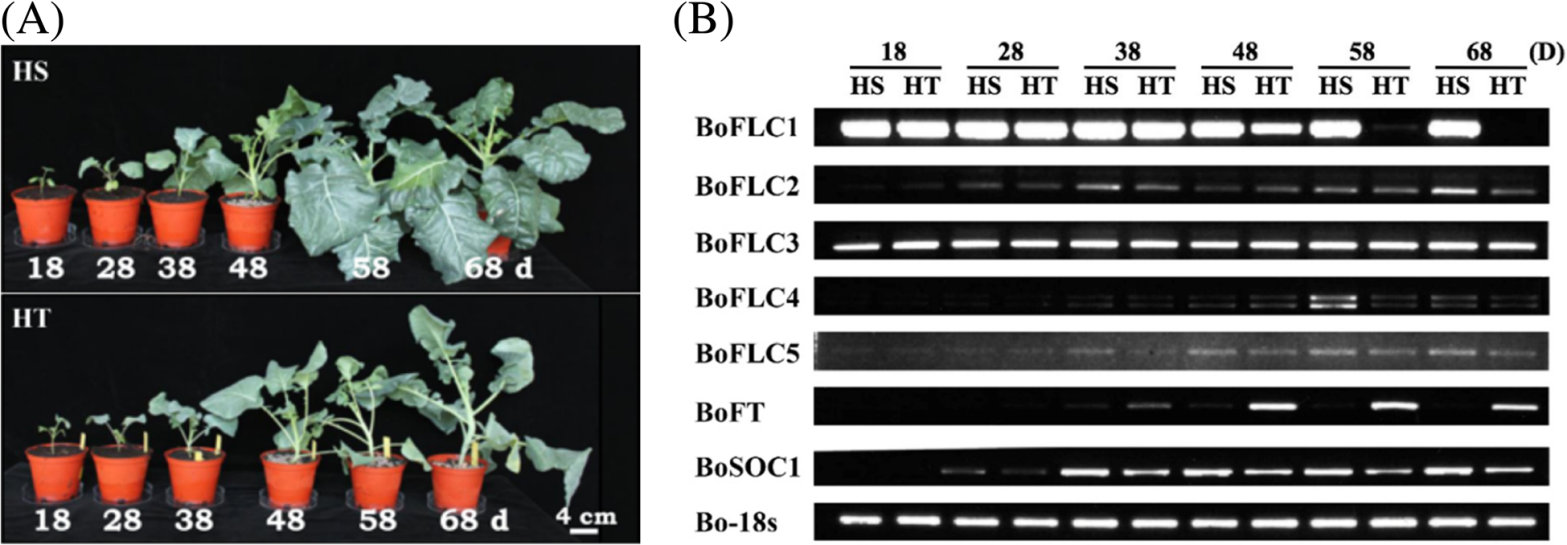
Growth and the expression of flowering-associated genes in the heat-tolerant and heat-sensitive broccoli genotypes. **a** Growth of the two broccoli lines at 22 °C at different times post germination (18, 28, 38, 48, 58 and 68 days). d, day. **b** RT-PCR analysis of flowering-associated genes at different growth stages in two broccoli genotypes. The cDNA sequences of *BoFLC1–5*, *BoFT*, *BoSOC1* and *Bo18S* with their corresponding accession numbers were obtained from GenBank of National Center for Biotechnology Information (Additional file 9: Table S7). The primers were designed to amplify these gene transcripts specifically. *Bo18s* was used as an internal control for RT-PCR analysis. White bar equals 4 cm

Previous work showed that only the HT genotype displayed head-forming capacity at temperatures up to 22 °C and 27 °C at 130 DPG (days post-germination) [37]. We aimed to evaluate the gene expression before the formation of the head in broccoli under high temperatures. The HT and HS genotypes were grown at 22 °C in long-day conditions with 18-h light / 6-h dark for 18, 28, 38, 48, 58 and 68 DPG (Fig. 4a). We found no floral organ primordia at 68 DPG in HT and HS broccoli. To determine the differential gene expression profiling of the flowering-development associated genes, total RNA was extracted from the shoot meristems in HT and HS broccoli and subjected to semi-quantitative RT-PCR. At 48, 58 and 68 DPG, *BoFLC1* was significantly downregulated in HT broccoli (Fig. 4b). The expression of *BoFLC2* was lower in HT than HS broccoli at 68 DPG. There were no significant changes of the expression level of other FLC homologs. *BoFLC1*, *2*, *3*, *4* and *5* genes were expressed to a similar level at 38 DPG between HT and HS genotypes, which suggests that both genotypes were at the same developmental stage. Taken together, these results and our previous study [37] indicate changed head-forming capacity under different temperature in the two broccoli genotypes. *BoFT* was predominately expressed in HT rather than HS broccoli.

### Expression profiles of high temperature-associated signaling components in HT and HS broccoli

**Fig. 5:**
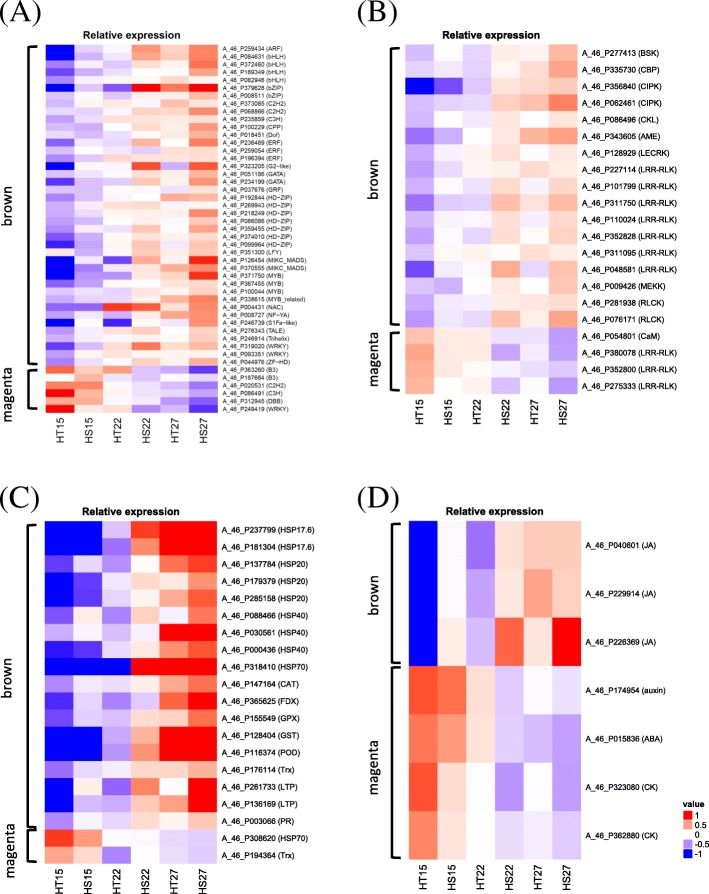
Heatmap of stress-associated signal component expression in the brown module. **a** Heatmap of genes encoding stress-associated signal proteins, including calmodulin (CaM), calmodulin binding protein (CaMBP), CBL-interacting protein kinase (CIPK), mitogen-activated protein kinase kinase kinase (MEKK), and calmodulin binding protein (CBP). **b** Heatmap of genes encoding HSP70 and HSP20 gene family. **c** Heatmap of genes encoding ROS homeostasis-associated proteins, including peroxidase (POD), thioredoxin (TRx), ferredoxin (FDX), catalase (CAT), and glutathione peroxidase (GPx). **d** Heatmap of genes encoding phytohormone-associated proteins, including auxin, cytokinin, and jasmonate

Perception and transmission of environmental signals are important for transition from vegetative to reproductive development. The brown module included 45 annotated TFs, which are classified into the ARF, bHLH, bZIP, C2H2, C3H, CPP, Dof, ERF, G2-like, GATA, GRF, HD-ZIP, LFY, MIKC-MADS, MYB, NAC, NF-YA, S1Fa-like, Trihelix and WRKY families (Fig. 5a and Additional file 4: Table S3). The expression of these TFs was significantly higher in HT and HS genotypes at 27 °C than 15 °C (*P* = 5.5e^− 11^, t-test). At 22 °C, these TFs showed a different expression pattern. Their expression was lower in the HT than HS genotype at 22 °C (*P* = 0.0001, t-test). Furthermore, the expression of another MADS-box TF, *AGL79*, was similar to the pattern of *FLC* in the brown module. Within the signaling components in the brown module, the expression of BR-signal kinase (BSK), LAMMER-type protein kinase (AME), lectin receptor kinase (LECRK), leucine-rich-repeat receptor-like kinase (LRR-RLK), receptor-like cytoplasmic kinases (RLCK), calmodulin (CaM), calmodulin binding protein (CaMBP), CBL-interacting protein kinase (CIPK), mitogen-activated protein kinase kinase kinase (MEKK), and calmodulin binding protein (CBP) was significantly higher in both HT and HS genotypes at 27 °C than 15 °C (Fig. 5b, *P* = 1.2e^− 08^, t-test). The expression of these genes was lower in HT than HS broccoli at 22 °C (*P* = 8.5e^− 05^, t-test).

To further our understanding of the stress response in broccoli to high temperature, we analyzed genes coding for HSPs, pathogenesis-related (PR), and ROS homeostasis-associated genes. The brown module of the eigengene expression profiles with increasing temperature had 17 *HSPs* (Fig. 5c). In both HT and HS broccoli, these *HSPs* were expressed higher at 27 °C than 15 °C. At 22 °C, the expression of these *HSPs* was lower in the HT than HS genotype. On the basis of the head-forming capacity in the previous study [37] and the expression of *HSPs* in this study, HT broccoli might be less sensitive to high temperature than HS broccoli. ROS homeostasis-associated genes, including peroxisomal catalase (CAT), ferredoxin (FDX), glutathione peroxidase (GPx), peroxidase (POD), and thioredoxin (Trx), were expressed higher in HT and HS genotypes at 27 °C than other temperatures (Fig. 5c). At 22 °C, the expression of ROS homeostasis-associated genes was lower in HT than HS broccoli. The expression of genes involved in the jasmonate (JA) metabolic pathway was changed by increasing temperature (Fig. 5d): it was higher in the two genotypes at 27 °C and 22 °C than 15 °C. Of note, genes involved in abscisic acid (ABA), auxin, and the cytokinin metabolic pathway were included in the magenta module. The expression patterns of auxin-, cytokinin- and ABA-related genes differed from that of JA in the brown module. Thus, the regulation of temperature-associated signaling components, defense genes, and phytohormones may be associated with head-forming capacity in broccoli under high temperature.

### Enrichment of gene ontology in HT and HS broccoli under high temperature

**Fig. 6:**
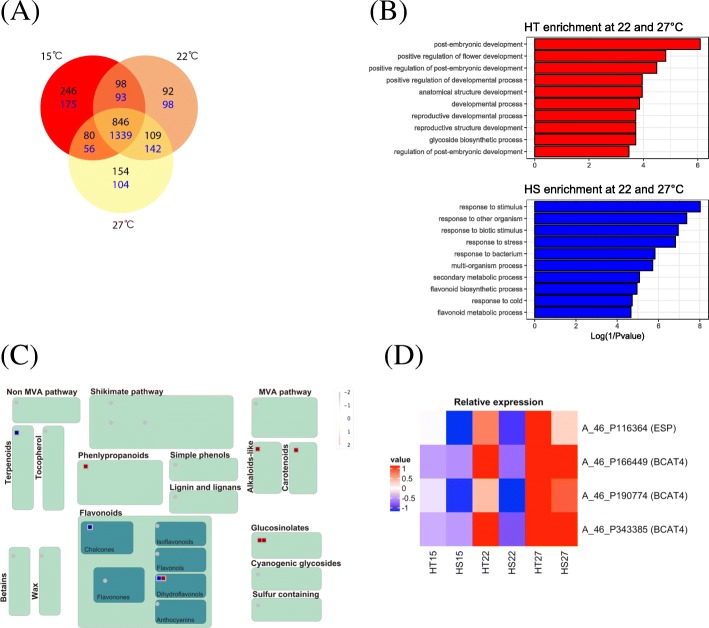
Distribution and functional classification of the differentially expressed genes. **a** Venn diagram showing the number of upregulated genes (numbers in black) in the HT genotype and the upregulated (numbers in blue) in the HS genotypes at 15 °C, 22 °C, and 27 °C. Results were based on the FDR < 0.05 and two-fold change in expression. **b** Gene Ontology analysis of the significantly temperature-associated genes (fold change ≥2) by microarray analysis. The agriGO database was used to perform enrichment analysis and the negative log of the P value is shown for the significantly enriched gene categories. **c** MapMan analysis of the secondary metabolism-associated genes. Each gene displayed as a square, red for upregulation and blue for downregulation. **d** The relative expression of probes assigned as epithiospecifier protein (ESP) and branched-chain aminotransferase4 (BCAT4) at 15 °C, 22 °C, and 27 °C in the HT and HS genotypes

To distinguish the differential effects of high temperature on HT versus HS broccoli, the gene differential expression profiles of the two broccoli genotypes were analyzed (Fig. 6a and Additional file 5: Table S4). After data were normalized, 1625 genes were found upregulated (fold change ≥2, HT/HS; *P* < 0.05, t-test) by at least one temperature exposure in HT broccoli and 2007 genes were upregulated in HS broccoli (fold change ≥2, HS/HT; *P* < 0.05, t-test). In total, 1189 genes at 27 °C and 1145 genes at 22 °C were expressed at significantly higher levels in HT than HS broccoli (*P* < 0.05, t-test). Functional annotation analysis of the microarray results involved using agriGO functional enrichment analysis (Additional file 6: Table S5). Among the HT-enriched genes at 22 °C and 27 °C, the top five significant GO terms were related to “post-embryonic development”, “positive regulation of flower development”, “positive regulation of post-embryonic development”, “anatomical structure development”, and “positive regulation of developmental process” (FDR adjusted *P* < 0.05; *P* < 0.05) (Fig. 6b). GO terms such as “response to stimulus”, “response to other organism”, “response to biotic stimulus”, “response to stress”, and “response to bacterium” were markedly over-represented in HS-enriched genes at 22 °C and 27 °C (Fig. 6b). These enriched functional categories in the HT genotype were mostly associated with developmental regulation. The enriched functional categories in the HS genotype were related to defense response, indicating differential physiological processes in response to high temperature between HT and HS.

### Distinct expression patterns of genes related to nucleotide and glucosinolate metabolism between HT and HS broccoli under high temperature stress

These results were further analyzed by comparison of metabolic genes using MapMan [45]. Only genes assigned to The Arabidopsis Information Resource (TAIR) locus were included in the MapMan analysis. Genes regulated by high temperature (22 °C and 27 °C) (fold change ≥2, HT/HS, *P* < 0.05, t-test) showed two functional enrichments (MapMan bins), including “nucleotide metabolism” (2 genes, *P* = 0.034) and “secondary metabolism” (9 genes, *P* = 0.045) (Additional file 7: Table S6). Genes involved in the “nucleotide metabolism” bin were apyrases protein (AYP5) and N-acetylglutamate kinase (NAGK). An analysis of “secondary metabolism” sub-bins revealed more detailed insights into plant secondary metabolism processes in broccoli. Two of nine genes were involved in “secondary metabolism sulfur-containing glucosinolates” and encode epithiospecifier protein (ESP) and branched-chain aminotransferase 4 (BCAT4). ESP catalyzes the formation of epithionitriles instead of isothiocyanates during glucosinolate hydrolysis. The methionine chain elongation cycle of aliphatic glucosinolate formation is mediated by BCAT4. The expression of *ESP* and *BCAT4* was upregulated in the HT genotype at 22 °C and 27 °C (Fig. 6c). One probe set was assigned as ESP and three as BCAT4 in the microarray data, which were significantly upregulated under elevated temperature conditions (Fig. 6d). The expression of *ESP* and *BCAT4* was low at 15 °C and high at 27 °C in both HT and HS broccoli. *ESP* and *BCAT4* were expressed at a higher level at 22 °C and 27 °C in the HT than HS genotype. Thus, regulation of genes responsible for glucosinolate metabolism may relate to head-forming capacity in HT broccoli.

### BoFLC1 is a candidate biomarker for heat-tolerant broccoli

**Fig. 7:**
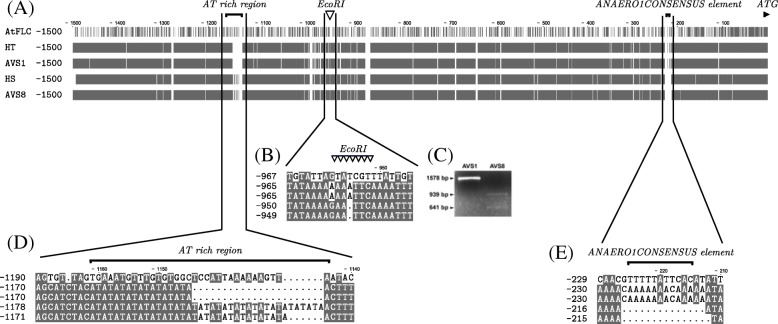
Broccoli *FLC1* promoter architecture. The sequences spanning the promoter region upstream of the start codon (− 1500 bp) were obtained by PCR using gene-specific primers. The PCR products were cloned into a TA-vector and 10 individual clones per genotype were sequenced. **a** Comparison of the *FLC1* promoter sequences of *Arabidopsis* and broccoli genotypes. The distinct conserved motifs in the promoter regions are marked with vertical black lines. The gray boxes indicate identical nucleotides. **b** The *Eco*RI restriction enzyme site is denoted with triangles. **c** Validation of *Eco*RI recognition site by restriction enzyme digestion of PCR amplicons and gel electrophoresis analysis. **d** The AT-rich region is denoted with a black line. **e** The ANAERO1CONSENSUS element is denoted with a black line

To assess the relationship between *BoFLC1* promoter and head development, we isolated the sequence upstream of the ATG codon (Fig. 7a). Four broccoli genotypes were selected to compare the variation of their promoter sequences. HT and AVS1 were heat-tolerant genotypes while HS and AVS8 were heat-sensitive genotypes. The *AtFLC* (*A. thaliana FLC*, AT5G10140) was included as a reference. Sequence alignment showed an *Eco*RI restriction site (GAATTC) that only existed within the promoter region of the HS and AVS8 genotypes (Fig. 7b). To test a restriction enzyme-based marker, a restriction enzyme digestion analysis was performed on PCR amplicons of the region. Indeed, digestion of the amplicon from the HS genotype with *Eco*RI resulted in two DNA fragments. One DNA fragment was observed in the HT genotype (Fig. 7c). Of note, we found differences in AT dinucleotide repeats and ANAERO1CONSENSUS element between the HT and HS genotypes (Fig. 7d and e). We found eight AT dinucleotide repeats in the HT and AVS1 genotypes. At least 16 AT dinucleotide repeats were detected in the HS and AVS8 genotypes (Table 1). The ANAERO1CONSENSUS sequence is usually present in promoters of anaerobically induced genes involved in the fermentative pathway [46]. The HT but not the HS genotype showed an ANAERO1CONSENSUS element. Amplicon sequencing analysis of the *BoFLC2* and *BoFLC3* promoter regions between the HT and HS genotypes were performed but the restriction fragment length polymorphisms were not found.

**Table 1:** Analysis of *BoFLC1* promoter DNA architecture in heat-tolerant and heat-sensitve broccoli

	genotype	AT dinucleotide repeat units
Heat-tolerant	B295 (HT)	8
	AVS1	8
	AV515	8
Heat-sensitive	BR1 op (HS)	19
	AVS8	16
	WF	16

According to the specific restriction enzyme site and the motif variations in the *BoFLC1* promoter between heat-tolerant and heat-sensitive genotypes, we designed PCR primers corresponding to their respective genomic DNA sequences. Primer set 1 specifically amplified the genomic DNA of HT broccoli (Additional file 8: Figure S2). Primer set 2 amplified the genomic DNA of HS broccoli. The method for detecting heat-tolerant broccoli genotyping was patented in Taiwan [47]. These variations suggest the potential of *BoFLC1* as a DNA biomarker for genotyping the HT and HS broccoli.

## Discussion

Broccoli is usually grown for its floral head production in regions where the average temperature is below 18 °C. When the temperature is higher than 27 °C, the head-forming capacity is significantly reduced or aborted. To overcome the high temperature effects, an inbred HT line (B295) was selected from Chinglong Seed Co. in Taiwan [36]. The HT proceeds with head formation under higher temperature (22 °C or 27 °C); however, the HS (BR1) continues vegetative growth. In this study, we investigated the gene expression profiles correlated with the head-forming capacity of two broccoli genotypes (HT and HS). The difference in expression patterns between HT and HS broccoli might lead to the identification of genes and pathways that may be important in the floral development of HT broccoli.

FLC is well-known as a repressor in flower development [3, 11]. The activated FLC represses downstream FT and SOC1 in the leaf and meristem, thereby acting as a repressor to delay flowering. Late-flowering ecotypes of *Arabidopsis*, such as Pitztal (Pit) and San Feliu-2 (Sf2) exhibited a higher expression of *FLC*. By contrast, late-flowering ecotypes Landsberg erecta (Ler) and Columbia (Col-0), showed a relatively lower level of *FLC* expression [48, 49]. Okazaki et al. (2007) [50] identified 5 *FLC* homologues from *B. oleracea* var. *italica* cv. Green Comet. In *B. oleracea* var. *botrytis*, *BoFLC2* has been found involved in reproductive development, and the transcription level was decreased by vernalization [51]. According to the transcriptional analysis in the present study, the expression of *BoFLC1* was significantly higher in the HS than HT genotype at temperatures up to 22 °C and 27 °C (Fig. 4). At 15 °C, the expression of *BoFLC1* was lower in both genotypes. The expression of *BoFLC2*, *BoFLC3*, *BoFLC4*, and *BoFLC5* was similar in the HT and HS genotypes. The HS broccoli produced heads at 15 °C but not 22 °C and 27 °C. Therefore, the head-forming capacity of HT broccoli may be associated with the expression of *BoFLC1* but not other FLC homologues.

High-throughput technologies can simultaneously measure the expression of thousands of genes within a particular mRNA sample. However, the large number of genes and the complexity of biological networks greatly increase the challenges of comprehending and interpreting the resulting mass of data. In this study, we used WGCNA [38](Langfelder and Horvath, 2008), a widely-used method that finds modules of highly correlated genes. WGCNA has been used to identify functionally enriched modules in human brain regions [52], animals [53], and plants [54, 55]. WGCNA revealed the brown module was significantly correlated with the temperature trait, and *FLC* was included (Fig. 1). Within the brown module, genes encoding TFs, signal components, HSPs, PR, ROS homeostasis, and JA metabolism were differentially expressed between HT and HS genotypes at 22 °C. The expression of these genes was higher in HS than HT broccoli.

Heat stress affects the process and structure of various proteins, membranes, RNA species, and cytoskeleton structures in plants, causing membrane fluidity imbalance [56]. The disrupted steady-state flux of plant cells might cause the accumulation of some toxic substrates such as ROS. Plants reprogram their transcriptome, proteome, and lipidome to respond to these effects [57]. The role of calcium transients in response to heat treatment in plants has been well documented [58]. A *CaM3* knockout mutant in *Arabidopsis* showed impaired thermotolerance, whereas overexpression of *AtCaM3* significantly increased the thermotolerance [59]. Mitogen-activated protein kinase (MAPK) cascades play important roles in plants in response to multiple stresses, including heat and heavy metals [60, 61]. Kovtun et al. (2000) [62] reported that overexpression of mitogen-activated protein kinase kinase kinase (ANP1) enhanced the thermotolerance of tobacco. Previous studies have shown that membrane-localized kinases play important roles in sensing various environmental stimuli and transduce them to downstream signaling networks [63, 64]. In *Arabidopsis*, JA contributes to thermotolerance, as demonstrated by mutant plants defective in JA signaling pathway [65]. Here, we showed that heat-associated signaling genes involved in calcium signals, MAPK cascades, LRR-RLKs, and JA biosynthesis were differentially expressed between the HT and HS genotypes. The complex signaling pathways may participate in the heat-stress response, which in turn contributes to head formation and thermotolerance mechanisms in broccoli.

HSPs are responsible for protein folding, assembly, translocation, and degradation in cellular processes, stabilizing proteins and membranes, and can assist in protein refolding under conditions of stress [66]. The production of HSP is a necessary step in plant heat acclimation [67]. Transgenic rice with increased levels of HSP17.7 protein showed significantly increased thermotolerance and greater resistance to UV-B stress [68]. Several lines of evidence have indicated that FLC is correlated with a thermal-response flowering time pathway [9, 69]. The expression of *HSPs* was induced with increasing temperature in both HS and HT genotypes. At 22 °C, the expression of *HSPs* was significantly higher in HS than HT broccoli, similar to the expression pattern of *BoFLC1* (Fig. 4b). We found more HSP-related genes with higher expression in HS than HT broccoli. These data suggest that HT broccoli was less sensitive to heat.

Glucosinolates are secondary metabolites synthesized by plants. They contain sulfur groups and are present in numerous species belonging to the *Brassicaceae* family such as broccoli and cabbage. When the tissue of these plants is damaged, glucosinolates can be hydrolyzed by plant myrosinase or non-enzymatically to form primarily isothiocyanates and/or simple nitriles [70, 71]. In *Arabidopsis*, BCAT4 catalyzes the chain elongation pathway of Met-derived glucosinolate biosynthesis [72]. A glucosinolate-deficient mutant of *Arabidopsis* showed thermosensitivity after high temperature stimulation [73]. In *Brassica oleracea*, soil temperature was correlated with accumulation of glucosinolates [74, 75]. Several genes involved in the glucosinolate metabolite pathway were differentially expressed in HT and HS cabbage lines [76]. We found the expression of three *BCAT4* probe sets higher in HT than HS broccoli at 22 °C (Fig. 6). The transcript levels of *BCAT4* were higher in the HT than HS genotype at 22 °C. Secondary metabolites have been proposed to participate in signaling mechanisms and modulation of physiological events in response to environmental stresses [74]. Thus, glucosinolate might be a signaling molecule that enhances thermotolerance to promote head formation in HT plants. ESP is also responsible for the products of glucosinolate toward epithionitriles [70, 77]. Ectopic expression of *Arabidopsis ESP* in *E. coli* triggered the hydrolysis of glucosinolate [77], and heating decreased plant ESP activity [70]. In this study, the expression of broccoli *ESP* was higher at 27 °C than 15 °C in both HT and HS genotypes. The expression of *ESP* was higher in HT than HS broccoli at 22 °C. Thus, BCAT4 and ESP, involved in synthesis and degradation of glucosinolate metabolism, were highly expressed in HT. These results imply that the turnover processes of glucosinolates may be enhanced in HT versus HS broccoli. Thus, HT broccoli may show an elevated level of glucosinolate under high temperature to modulate physiological responses during head formation.

Plants live under a continuous threat from abiotic and biotic stresses and have evolved mechanisms to minimize the negative impact of these factors, including the development of physical and chemical defenses [78, 79]. Previous studies proposed that investing in defense can come at the expense of other types of growth [80, 81]. Our co-expression network analysis revealed that the HS genotype was enriched with transcripts involved in signal transduction and defense compounds as compared with the HT genotype. The expression of one of the highly connected genes in the magenta module belonged to the NADPH-dependent thioredoxin reductase (NTRC). NTRC was reported as a high-efficiency redox system and was shown to play a role in protection against oxidative damage [82]. The highly connected genes in the WGCNA module are considered key drivers of physiological processes. Therefore, the NADPH-dependent thioredoxin reductase might play an important role in floral head formation in HT genotypes.

We also found higher expression of ABA, auxin and cytokinin metabolism-associated genes at 15 °C and 22 °C in the HT than HS genotype. ABA is a stress hormone that regulates diverse physiological processes antagonistically such as plant growth. Auxin mediates diverse processes in plant development, such as induction of floral primordia and regulation of flower development [83, 84]. Cytokinins are involved in many aspects of plant growth and development. For example, cytokinin promotes plant flowering [85, 86]. In this work, the increased expression of genes related to auxin biosynthesis and cytokinin metabolism may have led to elevated levels of auxin and cytokinin and promoted head formation in the HT genotype. The expression of genes involved in the JA-mediated pathway was induced in both genotypes with increasing temperature (15–27 °C) (Fig. 6). By contrast, auxin- and cytokinin-related genes showed markedly opposed expression patterns. Auxin and cytokinin are vital to regulate plant growth and development, and JA is responsible for activation of stress responses [28]. Thus, regulation of hormone homeostasis may be correlated with head formation in broccoli at high temperature.

LRR-RLKs are the largest subfamily of RLK and contain an extracellular LRR domain. They consist of a distinct extracellular domain to specifically sense signals, a transmembrane domain, and an intracellular kinase domain. LRR-RLKs have been found to function as receptors for phytohormones, small peptides or pathogen-derived molecules to modulate plant growth, reproduction and defense responses [87]. Downstream of the LRR-RLKs, MAPK cascades are key signaling transduction modules that act through phosphorylation of different targets. LRR-RLKs are known to recognize pathogen-associated molecular patterns (PAMPs). LRR-RLK-mediated signaling are core modulators of growth-defense tradeoffs in response to pathogen attack [88]. Activation of the defense responses to pathogens generally comes at the expense of plant growth [28]. In this study, LRR-RLKs (54 interactions) were identified as a potential hub genes within the brown module (Fig. 3). We revealed a possible role for LRR-RLKs in optimizing the heat stress signaling (MAPKs) versus phytohormone-mediated reproduction programs during head formation in broccoli (Figs. 3 and 5). LRR-RLKs localize at the plasma membrane for the perception of endogenous or exogenous signals to regulate plant growth, development, and immunity. They are responsible for sensing and transducing environmental fluctuations into change in downstream gene expression [89]. Thus, the difference in head-forming ability in response to high temperature between HT and HS broccoli may be related to the differential LRR-RLK gene expression. Thus, LRR-RLKs may play a role in tradeoffs between survival and reproductive effort.

In addition, polymorphisms of *LRR-RLK* genes can be applied to marker-assisted selection for crop improvement. For example, a deletion affecting an *LRR-RLK* gene was associated with the flat shape trait in peach [90]. In this work, transcript analysis revealed differential expression of *LRR-RLK* genes between HT and HS broccoli (Fig. 3). Therefore, it is suggested that *LRR-RLKs* are candidate genes for breeding heat-tolerant lines with head-forming capacity.

The expression of *BoFLC1* differed between HT and HS broccoli under mild heat treatment (22 °C) (Fig. 4). The expression patterns of other *FLC* homologues in broccoli were similar in HT and HS broccoli. The nucleotide sequences of the *FLC* coding region of *Arabidopsis thaliana* Col-0, Ler, and C24 were identified [91]. The predicted amino acids of the Col-0, Ler, and C24 FLC alleles were identical. Thus, differential regulation of the *cis*-elements at the *FLC* alleles might occur in these *Arabidopsis* ecotypes. Sheldon et al. (2000a) [3] investigated the sufficiency of the promoter region of *FLC* in *Arabidopsis* for repression of flowering initiation. In this study, the AT-rich region and ANAERO1CONSENSUS element in the promoter region of *BoFLC1* differed between HT and HS broccoli. Changes in the number of AT dinucleotide repeat units in the yeast *SDT1* promoter may cause alteration in gene expression level [92]. The *SDT1* promoters with 13 to 16 AT dinucleotide repeat units increased gene expression as compared with those with 7 to 9 repeat units. In *Liriomyza sativae*, the AT-rich element in the promoter region of *HSP* genes contributed to their regulatory activity of *Hsp70* under heat treatment [93]. In this study, the *BoFLC1* promoter region showed a larger AT-rich region in HS than HT broccoli. This promoter structure may enhance *BoFLC1* expression and thus delay broccoli head formation. ANAERO1CONSENSUS, the additional promoter element, was identified only in HT broccoli. Previous studies indicated that the ANAERO1CONSENSUS element in *Arabidopsis* was related to anaerobic responses [46]. The role of ANAERO1CONSENSUS in flowering regulation requires more investigation.

In *Arabidopsis*, AP1 acted as one of the positive regulators of flowering development [94, 95]. In our previous study [37], the expression of *BoAP1* was greater in HT than HS broccoli at 120 days post-germination at 22 °C. The expression of *AP1* is activated by FT, and FLC represses the activation of FT [96, 97]. *BoFT* was expressed to a higher level in HT than HS broccoli at 50 days post-germination at 22 °C. The head formation of HT broccoli was observed during 90 days post-germination. The expression of *BoAP1* and *BoFT* genes indicated the initiation of flower primordia.

## Conclusions

We provided gene expression profiles and pathways that might be associated with head-forming capacity in broccoli under high temperature. The suppression of *BoFLC1* expression in HT broccoli suggested the transcriptional regulation of the head-forming capacity in this genotype. From expression pattern of high-temperature-associated signaling genes, HS broccoli may invest in coping with stress instead of transition to the reproductive phase in response to high temperature. The differences in the *BoFLC1* promoter structure between HT and HS genotypes may be served as a DNA marker for genotyping of HT broccoli. Accordingly, high-resolution melting curve-based analysis can be developed for rapid and precise detection of the two genotypes. Our previous study showed that the AVS1 (HT1) had a similar phenotype as the HT line with normal head formation under high temperature as compared with the HS line [37]. The expression of *BoFLC1* was lower in the AVS1 than HS genotype. Thus, lowering *BoFLC1* expression could confer head-forming capacity under heat stress. The expression of glucosinolate metabolic-associated genes was higher in HT than HS broccoli. Transcriptome profiling of the HT and HS broccoli not only helps to understand the molecular mechanisms underlying head-forming capacity but also has potential for marker-assisted genotyping.

## Methods

### Plant materials

The heat-tolerant *Brassica oleracea var. italica* B295 (assigned as HT broccoli), and heat-sensitive *B. oleracea* var. *italica* BR1 op (assigned as HS broccoli) were provided by Ching Long Seed Co. The HT genotype, which is a heat-tolerant inbred line [36], exhibits head-forming ability under relatively high temperature (27 °C). Heat-sensitive broccoli cannot yield floral heads at higher temperatures. AVS1 and AV515, heat-tolerant genotypes, and AVS8 and WF, heat-sensitive genotypes, were provided by The World Vegetable Centre (AVRDC, Taiwan). Heat-tolerant AVS1 is a recombinant inbred line derived from two parental strains, BRS01 and BRS57 [98]. The HT and HS seeds (*n* = 20) were germinated in vermiculite for 10 days at 22 °C and transplanted to 9 cm diameter pots containing soil for another 30 days before treatment. HT and HS plants were transferred to elevated temperature conditions (15 °C, 22 °C, and 27 °C) for another 10 days. All plant materials were kept in growth chambers under 18 h light/6 h dark conditions. Shoot meristem from 50-day-old plants before head formation were collected and immediately frozen in liquid nitrogen. Three biological replicates were performed with consistent data.

### DNA extraction

Broccoli genomic DNA was extracted by DNeasy Plant Mini Kit (Qiagen, Hilden, Germany). A total of 100 mg of pooled leaf tissue was collected and ground to a fine powder in liquid nitrogen according to the protocol of the DNeasy Plant Mini Kit. The DNA was eluted in 50 μl sterilized RNase-free water. DNA quality and concentration was determined using a NanoDrop 2000 (Thermo Fisher Scientific, Wilmington, DE, USA).

### RNA extraction

Samples were isolated from the entire shoot meristem, including three individual plants. Total RNA was extracted with RNeasy Plant Mini Kit (Qiagen, Hilden, Germany). The RNA was further treated with DNase to remove DNA contamination (Qiagen, Hilden, Germany). The RNA samples were purified with the RNeasy MinElute Cleanup Kit (Qiagen, Hilden, Germany). Total RNA quality and concentration was determined by measuring the ratio of A260/A280 and A260/A230 using NanoDrop 2000 (Thermo Fisher Scientific, Wilmington, DE, USA). RNA samples of more than 2 μg/μl concentration and high purity (OD260/280 > 2, OD260/230 > 2) were used for semi-quantitative RT-PCR and microarray assay.

### Semi-quantitative RT-PCR

First-strand cDNA was synthesized from 2 μg of total RNA with 2.5 μM oligo(dT)15 primers by using the Improm-II reverse transcription system (Promega, Madison, WI, USA). 18 s rRNA was used as an internal control. The PCR cycling involved an initial denaturation step at 94 °C for 2 min, 27–40 cycles of amplification and a final elongation step at 72 °C for 5 min. PCR products were analyzed on a 1% (*w*/*v*) agarose gel. Three biological replicates were performed for each gene expression analysis. One of three biological replicates is represented. The primer sequences are listed in Additional file 9: Table S7.

### Microarray analysis

Gene expression quantification was performed with a Brassica Gene Expression Microarray containing 4x44k probe sets on a single chip (Agilent, Cat. No. G2519F-022520, Palo Alto, CA, USA). Labelling, hybridizations, and data analysis (one sample per chip) were carried out according to the manufacturer’s manual. The trimmed mean target intensity of each array was arbitrarily set to 100. Raw cell intensity data files were imported into Genespring software v12 (Agilent Technologies, Inc., Palo Alto, CA, USA). The data were normalized with the Robust Multichip Average (RMA) algorithm on the basis of median baseline and converted to log2 scale to allow the comparison of the three biological replicates of HT and HS under 15 °C, 22 °C and 27 °C treatments. Significantly different gene expression was detected on the basis of the *t* test. The Benjamini and Hochberg algorithm was used for adjusting *P* values. Genes were considered as significantly up- and down-regulated if the FDR-adjusted *P* value for the corresponding probe set was < 0.1. The microarray data described in this study have been deposited in Gene Expression Omnibus (series accession no. GEO: GSE97528) (https://www.ncbi.nlm.nih.gov/geo/query/acc.cgi?acc=GSE97528).

### Co-expression network analysis

Gene co-expression network analysis was performed using the R package WGCNA (version 1.51) [38]. Probe sets with a fold change ratio < ±1.2 in HT genotype relative to HS and FDR adjusted *P* > 0.1 in any of the 18 microarray samples were excluded. The co-expression analysis was started by calculating a correlation matrix containing all pairwise Pearson correlations between all pairs of probes across all selected microarray samples. We used signed network and soft-thresholding power (β = 8) to obtain an adjacency matrix. Then the adjacency matrix was transformed into a topological overlap matrix (TOM) with the topological overlap (TO)-based dissimilarity (1-TOM) [99]. This step resulted in a clustering tree (dendrogram) whose branches were identified for cutting depending on their shape using the dynamic tree-cutting algorithm [100]. Modules were defined as branches of the dendrogram obtained from clustering and labeled by colors beneath the dendrogram. The first principal component of each module was defined as the module eigengene (ME), which could be considered a weighted average of the gene expression profiles that made up the module. The module membership (MM) was defined as the correlation of expression profile and each ME. To discover any significant relationships between gene expression perturbations within modules and traits, we computed the correlations between MEs and stress stimulations. *P* values were obtained via the Fisher transformation of each correlation.

### Gene functional categorization

The differentially expressed genes were manually classified based on information from the following resources: gene ontology data according to agriGO GO analysis toolkit [39], gene classification from MapMan software 3.5.1 [45], and a literature search using singular enrichment analysis (SEA) with default parameters. For MapMan analysis, the averaged signals for a given treatment in one genotype were expressed relative to that of the other genotype, converted to a log2 scale and displayed. *A. thaliana* mapping files were imported into MapMan. Broccoli genes assigned with the TAIR locus were organized by bins and sub-bins for display on the schematic map. Gene expression was analyzed by the Wilcoxon Rank Sum test with an uncorrected *P* value. *P* < 0.05 was considered statistically significant.

## Additional files

Additional file 1: **Figure S1.** Expression of genes of WGCNA modules. (DOCX 1843 kb)

Additional file 2: **Table S1.** GO functional categories (biological process) of probes in the brown module. (XLSX 14 kb)

Additional file 3: **Table S2.** The calculation of gene interactions by network component analysis. (XLSX 92 kb)

Additional file 4: **Table S3.** Significantly regulated genes in the WGCNA modules. (XLSX 319 kb)

Additional file 5: **Table S4.** The HT and HS enriched gene sets. (XLSX 430 kb)

Additional file 6: **Table S5.** GO functional categories (biological process) of probes in the HT and HS enrichment gene sets. (XLSX 59 kb)

Additional file 7: **Table S6.** MapMan analysis results of the HT and HS enrichment gene sets. (XLSX 10 kb)

Additional file 8: **Figure S2.** The amplification of the template genomic DNA with specific primer sets. (DOCX 124 kb)

Additional file 9: **Table S7.** Primers used in PCR and RT-PCR in this study. (XLSX 10 kb)

## Abbreviations

AP1: APETALA1
AYP: Apyrases protein
BCAT4: Branched-chain aminotransferase 4
CAT: Catalse
CO: CONSTANS
DPG: Days post germination
ESP: Epithiospecifier protein
FDX: Ferredoxin
FLC: FLOWERING LOCUS C
FT: FLOWERING LOCUS T
GO: Gene ontology
GPx: Glutathione peroxidase
GS: Gene significance
HS: Heat-sensitive
HSP: Heat shock protein
HSR: Heat shock response
HT: Heat-tolerant
JA: Jasmonate
LFY: LEAFY
LRR-RLK: Leucine-rich repeat receptor-like kinase
MAPK: Mitogen-activated protein kinase
ME: Module eigengene
MM: Module membership
NAGK: N-acetylglutamate kinase
POD: Peroxidase
ROS: Redox oxygen species
SOC1: SUPPRESSOR OF OVEREXPRESSION OF CO 1
SOD: Superoxide dismutase
TRx: Thioredoxin
WGCNA: Weighted correlation network analysis
