# Supplementary figures and images for: Analysis of ambient temperature-responsive transcriptome in shoot apical meristem of heat-tolerant and heat-sensitive broccoli inbred lines during floral head formation
Source: BMC Plant Biol. 2019 Jan 3;19:3. doi: 10.1186/s12870-018-1613-x (PMC6318969; doi:10.1186/s12870-018-1613-x)

Fig. S1


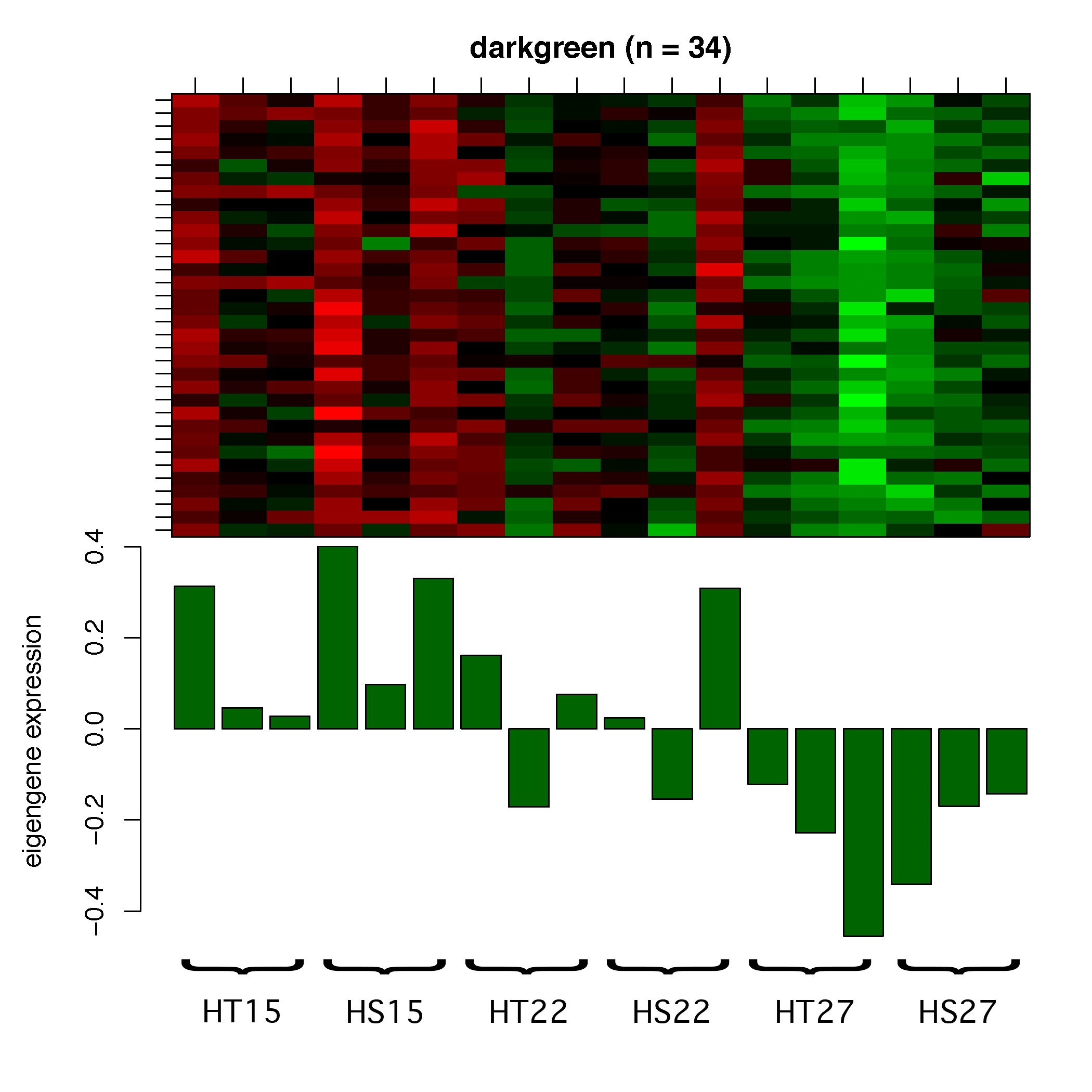

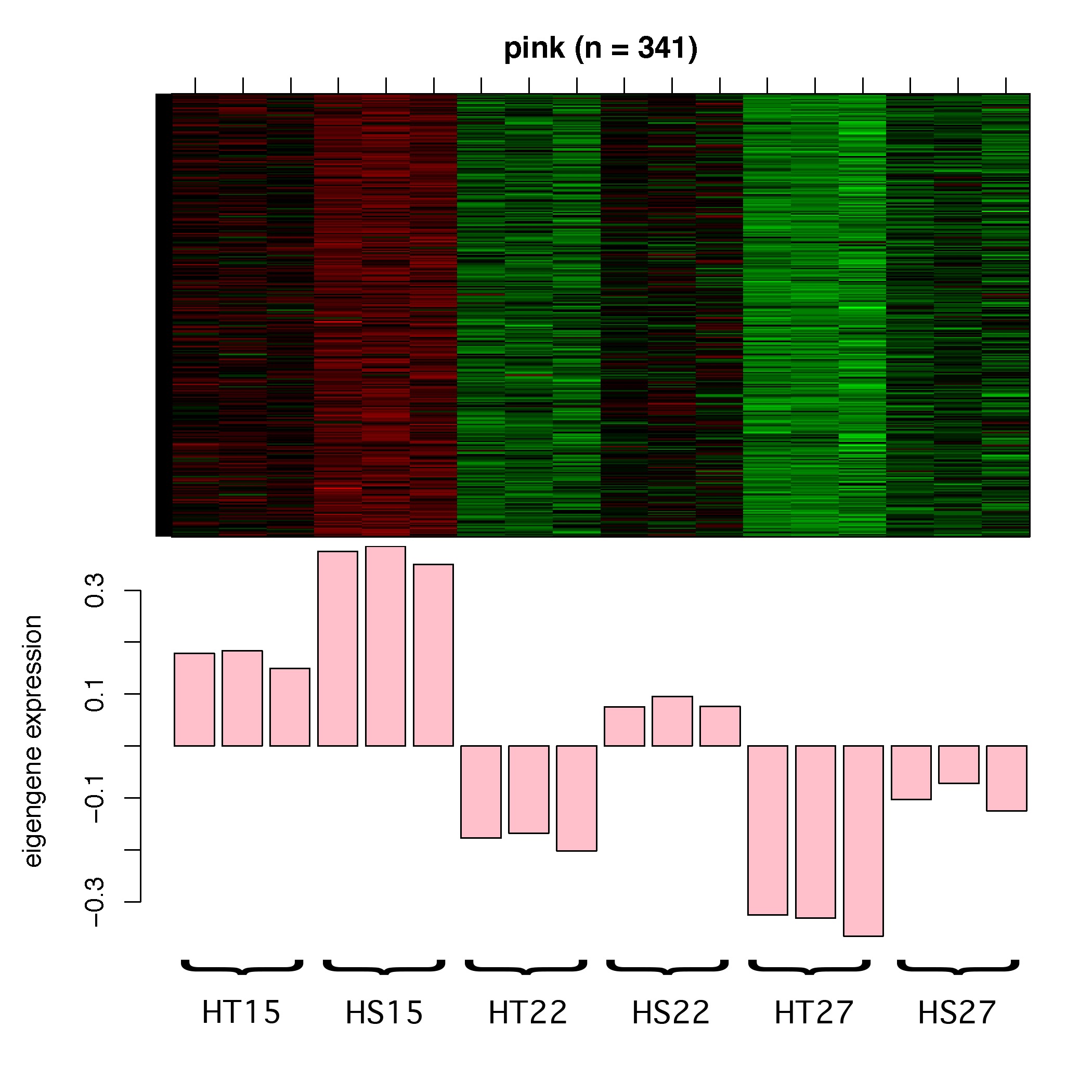


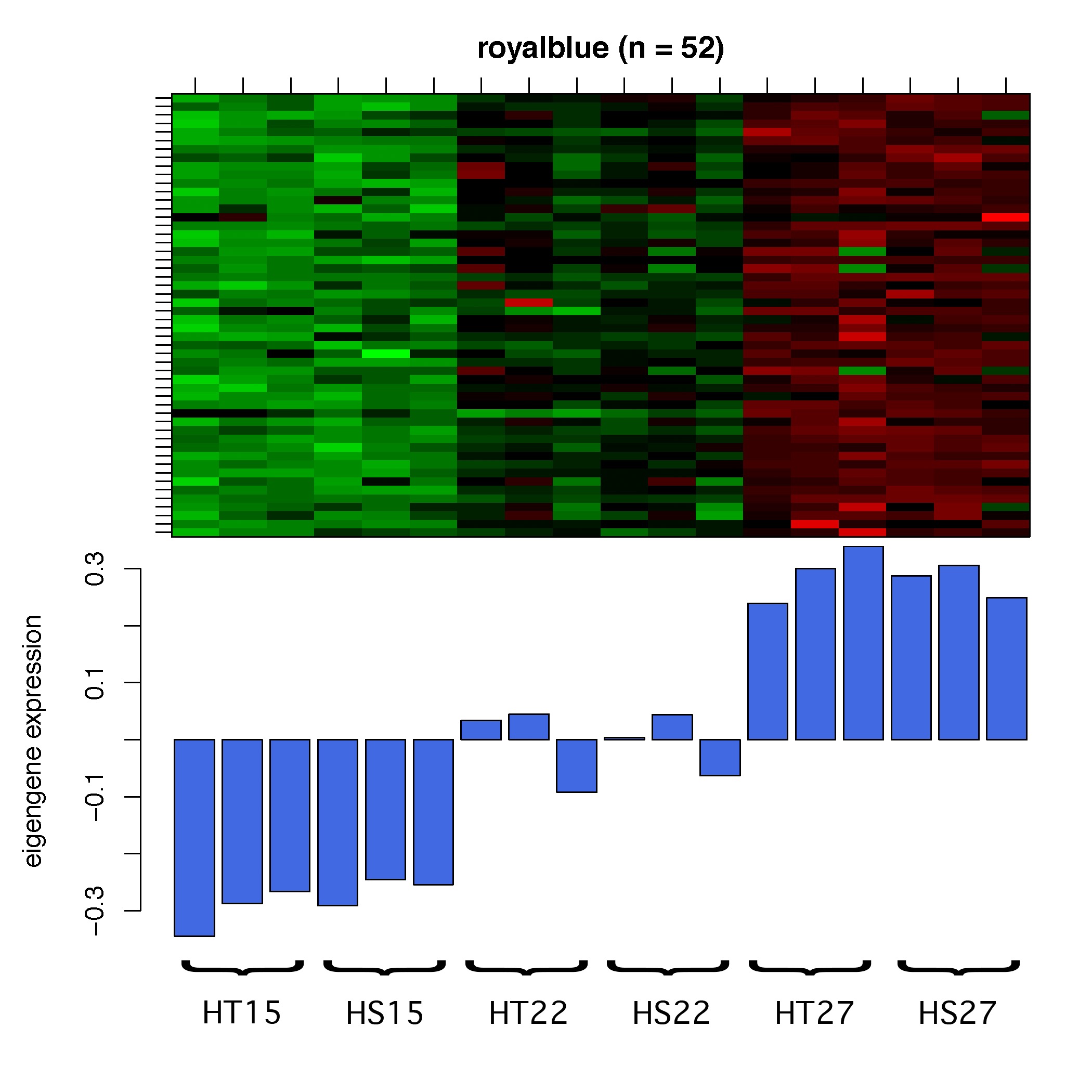

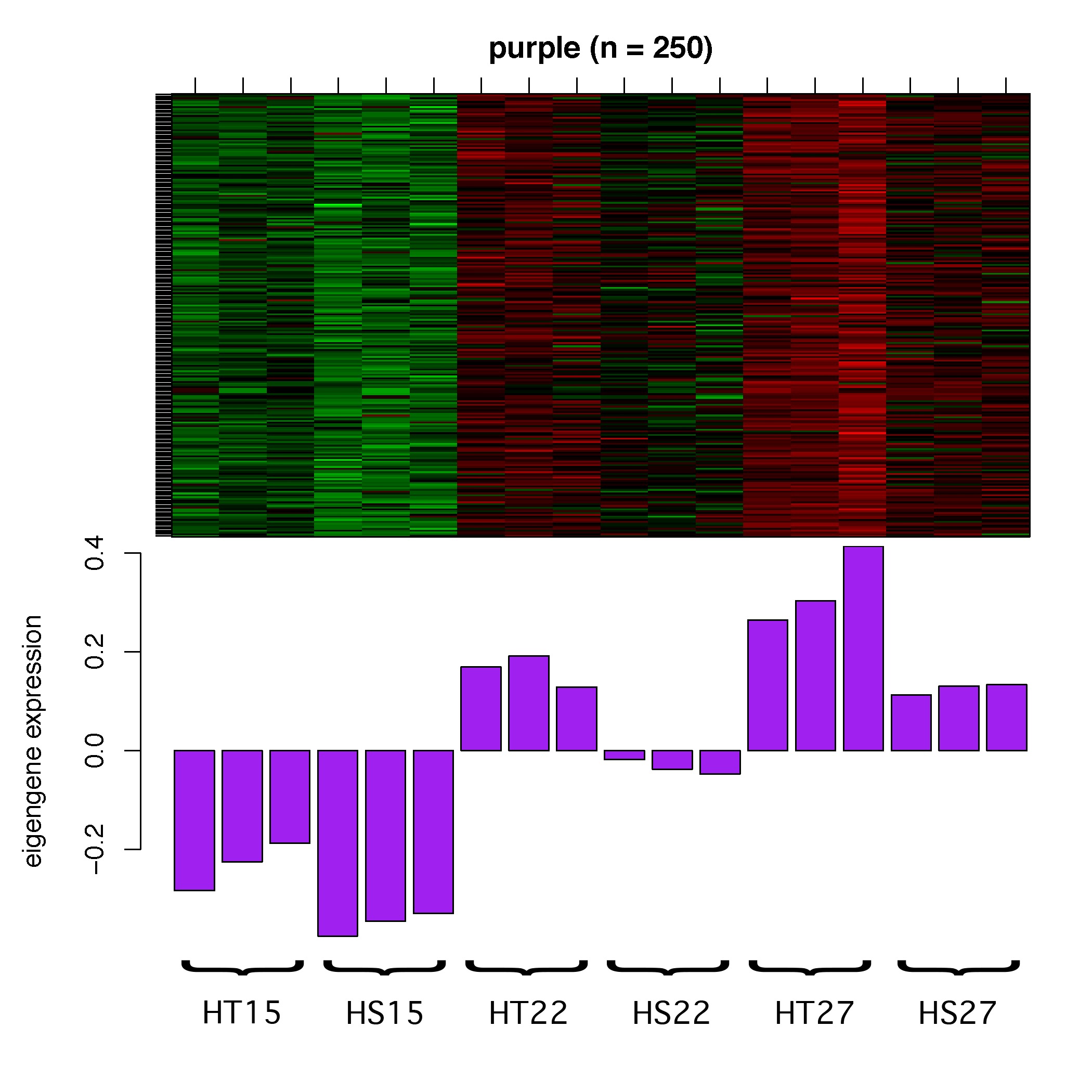

Supplement: Supplementary file 1 — Figure S1. Expression of genes of WGCNA modules. (DOCX 1843 kb) [file 12870_2018_1613_MOESM1_ESM.docx]

Fig. S2

HT HS HT HS F1

1872 bp

set 1


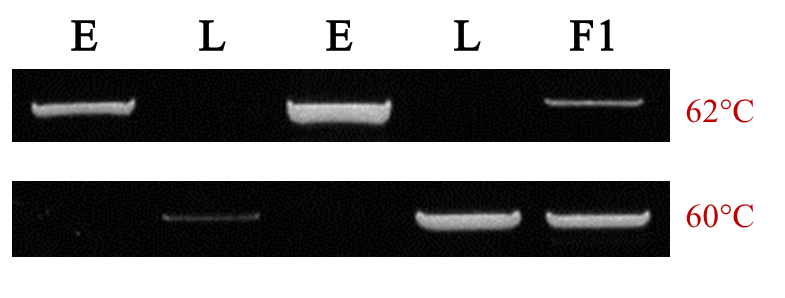


1859 bp

set 2

Supplement: Supplementary file 8 — Figure S2. The amplification of the template genomic DNA with specific primer sets. (DOCX 124 kb) [file 12870_2018_1613_MOESM8_ESM.docx]
